# Plastic Workability and Rheological Stress Model Based on an Artificial Neural Network of SiC_p_/Al-7.75Fe-1.04V-1.95Si Composites

**DOI:** 10.3390/ma17215317

**Published:** 2024-10-31

**Authors:** Pinming Feng, Shuang Chen, Jie Tang, Haiyang Liu, Dingfa Fu, Jie Teng, Fulin Jiang

**Affiliations:** 1 College of Materials Science and Engineering, Hunan University, Changsha 410082, China; pmfeng@hnu.edu.cn (P.F.); tangiie94@foxmail.com (J.T.); tengjie@hnu.edu.cn (J.T.); 2 Hunan Provincial Key Laboratory of Vehicle Power and Transmission System, Hunan Institute of Engineering, Xiangtan 411104, China; chens20200528@126.com; 3 Hunan Province Engineering Research Center for the Preparation and Application of High Performance Aluminum Matrix Composites, Luxi 416100, China; sale@hn-jm.com; 4 Hunan Everrich Composite Corp, Luxi 416100, China

**Keywords:** aluminum matrix composites, deformation behavior, ANN model, finite element simulation

## Abstract

SiC_p_/Al-Fe-V-Si composites exhibit complex deformation behaviors at both room and high temperatures because of the presence of SiC reinforcement particles and numerous fine dispersed Al_12_(Fe, V)_3_Si heat-resistant phases. In this work, an artificial neural network (ANN) constitutive model was established to study the deformation behavior of SiC_p_/Al-7.75Fe-1.04V-1.95Si composites over a wide temperature range based on uniaxial compression. Then, microstructural observation, finite element analysis, and processing maps were utilized to investigate the plastic workability. The results showed that the ANN model fit the experimental stress–strain curves with high accuracy, achieving an *R*^2^ value of 0.999. The ANN model was embedded into finite element software to study plastic deformation behaviors, which indicated that this model could accurately compute the plastic and mechanical response during the compressing process. Finally, a thermomechanical processing diagram was developed, revealing that the optimal processing parameters of the SiC_p_/Al-7.75Fe-1.04V-1.95Si composites were a deformation temperature of 450–500 °C and a deformation rate of 0.1–0.2 s^−^^1^.

## 1. Introduction

Aluminum matrix composites (AMCs) are highly favored because of their characteristics such as high specific strength, high specific modulus, wear resistance, and excellent high-temperature performance [1,2]. However, because of significant differences in physical and chemical properties between the reinforcement and aluminum matrix, aluminum matrix composites are difficult to process thermoplastically like aluminum alloys, resulting in huge challenges in practical applications [3]. The mechanical behavior of materials is correlated with their microstructures. The addition of reinforcements complicates the microstructural changes during the deformation of aluminum matrix composites. Improper processing can lead to issues such as material cracking, particle fracture, and interface debonding [4].

Regarding the deformation behavior of materials, researchers have described the deformation behavior of materials under certain conditions by establishing various constitutive equations. Commonly used constitutive models include the Arrhenius model [5], the Zerilli–Armstrong (ZA) model [6], and the Johnson–Cook (JC) model [7]. Constitutive equations can determine the relationship between flow stress and influencing factors such as strain and temperature, and they are mostly used for the hot deformation process. Wu et al. [8] developed a multi-sized SiC particle-reinforced 6013 aluminum matrix composite and investigated the high-temperature deformation behavior of the material by establishing an Arrhenius constitutive equation. They estimated the thermal deformation activation energy of the composites at different strain levels through the constitutive relationship, finding that this energy slightly increased with the increase in strain. However, the material deformation process is a highly complex nonlinear system. Using traditional constitutive models to fit deformation behavior is a cumbersome process, and experimental results are often subject to various random factors, leading to discrepancies between experimental values and predictions. Recently, machine learning technology has been applied as a new approach to predict the performance of materials [9]. N. Haghdadi et al. [10] used both an Arrhenius-type constitutive equation and artificial neural network (ANN) models to predict the high-temperature rheological behavior of cast A356 aluminum alloy. Their study showed that the trained ANN model was a powerful tool for predicting the high-temperature flow behavior of cast A356 aluminum alloy. In addition, Huang et al. [11] developed an optimized BP-ANN model based on a genetic algorithm (ANN-GA model) to predict the flow behavior of 5754 aluminum alloy. Their results indicated that the ANN-GA model had higher accuracy compared with the BP-ANN model.

Al-Fe-V-Si heat-resistant aluminum alloys were developed in 1986 by Skinner D.J. and others from Allied Signal using the semi-continuous casting method [12]. This alloy primarily relies on the fine dispersion of the Al_12_(Fe, V)_3_Si heat-resistant phase to achieve high-temperature strengthening. It features high strength at both room and elevated temperatures, good thermal stability, strong resistance to creep, high stiffness, and a slow coarsening rate of reinforcing phase [13]. When reinforcement particles are incorporated into Al-Fe-V-Si alloys, the presence of both the reinforcement particles and the numerous fine dispersed Al_12_(Fe, V)_3_Si heat-resistant phases makes the deformation behavior complex at both room and elevated temperatures. It is therefore necessary to study the deformation behavior of particle-reinforced Al-Fe-V-Si heat-resistant aluminum matrix composites over a wide range of temperatures.

Hence, this paper prepared heat-resistant aluminum matrix composites by introducing SiC particles into the Al-7.75Fe-1.04V-1.95Si heat-resistant aluminum alloy. Uniaxial compression experiments were conducted to obtain the flow stress–strain curves of SiC_p_/Al-7.75Fe-1.04V-1.95Si composites under different deformation parameters. An artificial neural network (ANN) model was established based on these data to predict the material’s deformation behavior and was successfully applied to finite element analysis (FEA). Finally, a thermomechanical processing diagram was established using the Dynamic Material Model to determine the “safe zone” for the thermal processing of SiC_p_/Al-7.75Fe-1.04V-1.95Si composites and to identify the optimal processing parameters.

## 2. Materials and Methods

**Figure 1:**
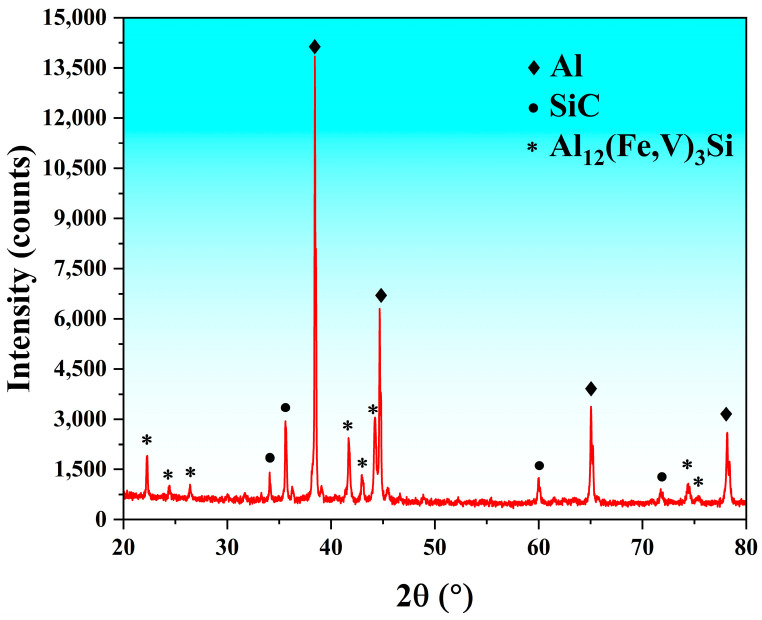
X-ray diffraction (XRD) pattern of SiC_p_/Al-7.75Fe-1.04V-1.95Si.

The SiC_p_/Al-7.75Fe-1.04V-1.95Si composites used in this experiment were prepared by powder metallurgy and hot extrusion processes. Specifically, 15 wt.% of SiC powder was uniformly mixed with Al-7.75Fe-1.04V-1.95Si alloy powder. The mixed powders were then cold pressed into billets under isostatic pressure, followed by vacuum hot pressing at 540 °C and 75 MPa for 4 h. The chemical composition of the Al-7.75Fe-1.04V-1.95Si alloy powder is shown in Table 1. To eliminate defects such as voids in the powder metallurgy-produced composite material, improve the interface bonding between the aluminum matrix and the reinforcement particles, and refine grain size, the aluminum matrix composites were subsequently hot extruded into rods. XRD analysis confirmed the presence of heat-resistant phase Al_12_(Fe, V)_3_Si in the prepared samples (Figure 1).

The hot extruded rods were processed into cylindrical compression specimens with a diameter of 8 mm and a height of 12 mm along the extrusion direction using electrical discharge machining (EDM). Uniaxial compression tests were conducted on a Gleeble 3500 thermal simulator. The experimental temperatures were 25, 100, 200, 300, 400, and 500 °C, with strain rates of 0.1, 1, and 10 s^−^^1^. All samples were finely polished to reduce processing defects. Graphite lubricant was applied between the sample and the die to minimize friction. The samples were heated to the desired deformation temperature at a rate of 10 °C/s, held for 300 s to ensure uniform temperature distribution, and then continuously compressed until a true strain of 0.5 was reached. The samples were immediately water-cooled to preserve the microstructure after hot compression.

The established artificial neural network model was integrated into ABAQUS using the Vuhard subroutine to develop a thermal compression finite element model. To reduce computational costs, the thermal compression model was configured as a quarter model. The upper and lower dies were set as rigid bodies, while the sample was modeled as an elastic–plastic material. Both the sample and die elements were set as C3D8RT elements. A Coulomb friction model with a friction coefficient of 0.3 was employed between the sample and the die. Three sets of thermal deformation parameters were defined for the thermal compression simulation. By comparing the load–displacement curves extracted from numerical simulations with experimental results, the reliability of the finite element model was validated.

## 3. Results and Discussion

### 3.1. Flow Stress

**Figure 2:**
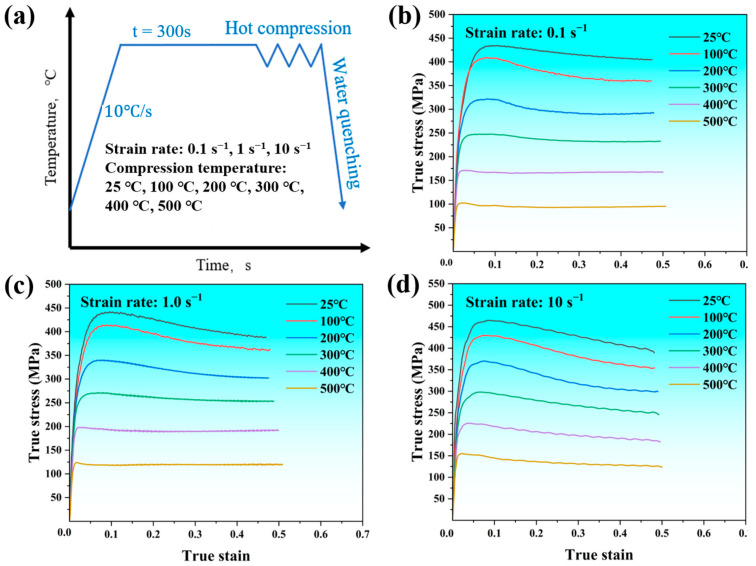
Compression stress–strain curves of the SiC_p_/Al-7.75Fe-1.04V-1.95Si composites in the temperature range of 25–500 °C and strain rate range of 0.1–10 s^−1^: (**a**) hot compression parameters and (**b**) 0.1 s^−1^; (**c**) 1 s^−1^; and (**d**) 10 s^−1^.

Figure 2 shows the true stress–true strain curves for the SiC_p_/Al-7.75Fe-1.04V-1.95Si composites at deformation temperatures ranging from 25 °C to 500 °C, with strain rates of 0.1, 1, and 10 s^−^^1^. The results indicate that, compared with most aluminum matrix composites [14,15], the SiC_p_/Al-7.75Fe-1.04V-1.95Si composites exhibit better high-temperature resistance. At a deformation temperature of 300 °C, the flow stress can be maintained around 220 MPa, and at 500 °C, it remains above 100 MPa. This is primarily due to the presence of a large amount of fine, dispersed Al_12_(Fe, V)_3_Si heat-resistant phase in the matrix. Additionally, the experimental results indicate that the rheological stress is significantly affected by the strain rate and deformation temperature, suggesting that the material’s deformation behavior is sensitive to deformation conditions. During the initial stage of strain, the stress increases rapidly with the increase in strain until it reaches the peak stress. At lower deformation temperatures, after the rheological stress reaches the peak stress, it decreases with increasing strain. This is likely due to the formation of cracks in the material during compression, ultimately leading to a reduction in rheological stress. In contrast, under high-temperature conditions, the rheological stress remains stable after reaching the peak stress. Additionally, under the same strain rate conditions, the peak stress of the composite material gradually decreases with increasing deformation temperature.

In fact, the deformation behavior of the composites is primarily due to the competition between work hardening and dynamic softening during the hot compression deformation process [16]. In the initial stage of deformation, because of the presence of SiC particles and nanoscale Al_12_(Fe, V)_3_Si dispersoids, a large number of dislocations during deformation are pinned down, leading to a sharp increase in true stress, exhibiting the phenomenon of work hardening. At lower deformation temperatures, work hardening is predominant. As the deformation temperature increases, the thermal motion and diffusion capability of atoms enhance, which may lead to softening phenomena such as dynamic recovery or dynamic recrystallization. This results in the reorganization and annihilation of dislocations within the material, leading to a decrease in dislocation density [17]. As a result, the rheological stress decreases gradually with increasing deformation temperature and remains stable after reaching the peak stress.

### 3.2. Microstructure Evolution

**Figure 3:**
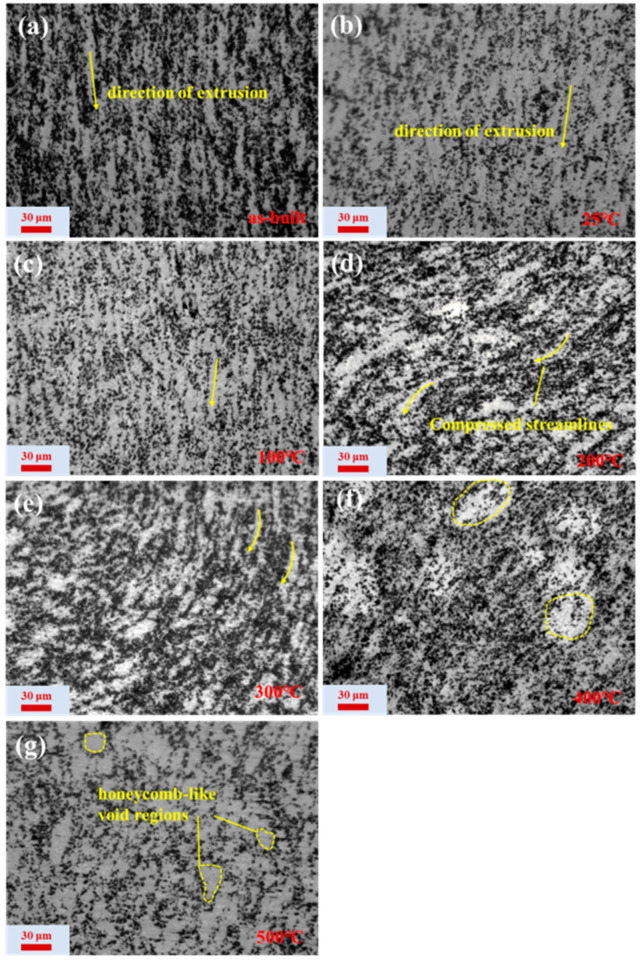
Microstructure of the SiC_p_/Al-7.75Fe-1.04V-1.95Si composites under different deformation conditions: (**a**) as-extruded sample; (**b**) 0.1 s^−1^-25 °C; (**c**) 0.1 s^−1^-100 °C; (**d**) 0.1 s^−1^-200 °C; (**e**) 0.1 s^−1^-300 °C; (**f**) 0.1 s^−1^-400 °C; and (**g**) 0.1 s^−1^-500 °C.

During plastic deformation, SiC particles can only deform by rotating or moving themselves to accommodate the material’s deformation. This leads to the redistribution of the reinforcement particles within the matrix. Different deformation parameters will affect the distribution of the particle reinforcement phase differently, ultimately influencing the overall performance of the material. Figure 3 presents the particle distribution along the longitudinal cross-section of the specimens under different deformation conditions. Figure 3a shows the microstructure of the sample before hot compression, where SiC particles are arranged in a streamlined pattern along the extrusion direction with no significant clustering. However, after compression, there is a notable change in the distribution of SiC particles in the sample. At a strain rate of 0.1 s^−1^, as the compression temperature increases, SiC particles transition from a streamlined distribution to a more disordered distribution. This change suggests that at higher deformation temperatures, the softening of the matrix alloy may facilitate easier rotation or movement of SiC particles, ultimately leading to their redistribution. Additionally, it can be observed that at a deformation rate of 0.1 s^−1^ and a deformation temperature of 500 °C, there are numerous honeycomb-like void regions devoid of SiC particles within the matrix (Figure 3g). This observation suggests that there may be some SiC particle streamlines with wider spacing in the sample, and shear deformation may further enlarge these void regions during compression deformation.

### 3.3. Artificial Neural Network Model

A constitutive equation is a mathematical model that describes the relationship between the deformation and stress of a material under external forces. Through constitutive equations, it is possible to predict and analyze the mechanical behavior of materials under different loading conditions. Johnson et al. [18] proposed an expression to predict the strain rate strengthening effect in metallic materials, known as the Johnson–Cook (JC) constitutive model. This model is widely used because of its simplicity and minimal number of parameters, but the stress curves predicted by this model do not match the measured stress curves very well. This is because the JC constitutive model only involves simple multiplication of strain, strain rate, and temperature without considering their coupling relationships, which limits the model’s applicability [19]. Therefore, most literature reports have modified the JC constitutive model to accommodate the deformation behavior of certain materials under specific conditions [20,21]. But this undoubtedly increases the workload and presents certain challenges.

To overcome the aforementioned drawbacks, artificial neural network (ANN) models, which do not require consideration of inherent physical deformation mechanisms or derivation of related mathematical models, have been successfully applied to predict the deformation behavior of metals and alloys [22,23]. An ANN consists of the following components: the input layer, hidden layer(s), and output layer [24].

This paper sets three nodes in the input layer, corresponding to the following influencing factors: strain, strain rate, and temperature. Research indicates that the number of nodes and layers in the hidden layer determines the accuracy of model training [25]. Therefore, the configuration of the hidden layers should be set according to the specific situation. The hidden layers process the data and pass the results to the output layer, ultimately providing the value of the flow stress *σ*. If the output layer fails to produce an accurate prediction, the model will perform backpropagation to transmit the error signal back to each neuron node. During this process, repeated iterative calculations are performed to ultimately achieve results with high prediction accuracy. Before training the model, it is necessary to normalize the input data so that their range is between 0 and 1. This is because the values of strain, strain rate, and temperature vary significantly, and without preprocessing, this can lead to difficulties in convergence and increased training complexity.

The neural network model in this paper is based on the work of Olivier Pantalé et al. [26]. During the model training process, two hidden layers were set up, with 15 and 7 nodes, respectively, in each layer, and the sigmoid function was used as the activation function (Equation (1)). The entire dataset in this paper consisted of 5400 data points, with 75% used for training and 25% used for testing. The artificial neural network training was implemented using Python. The accuracy of the trained model was precisely described using the coefficient of determination (*R*^2^), root mean square error (*RMSE*), and mean relative error (*MRE*) functions (Equations (2)–(4)).

**Figure 4:**
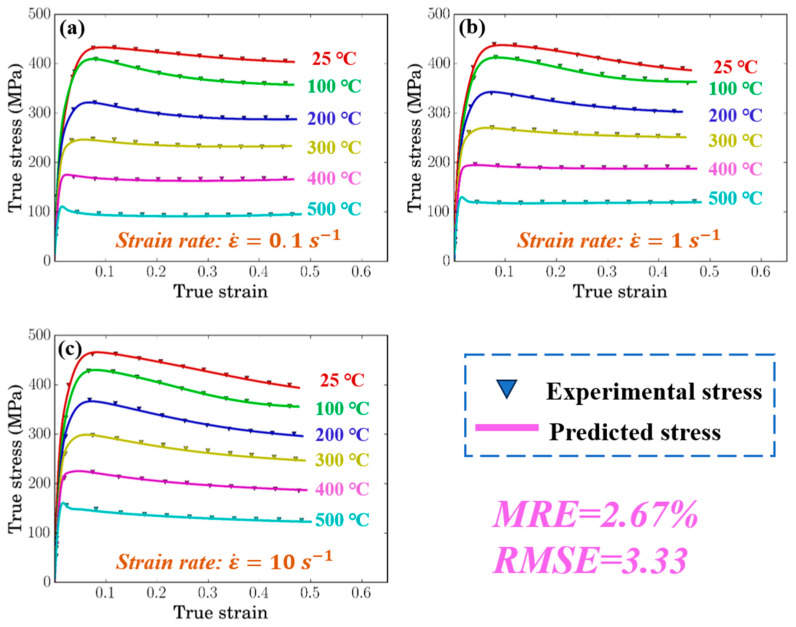
Comparison of experimental and calculated results at different strain rates: (**a**) 0.1 s^−1^; (**b**) 1 s^−1^; and (**c**) 10 s^−1^.

**Figure 5:**
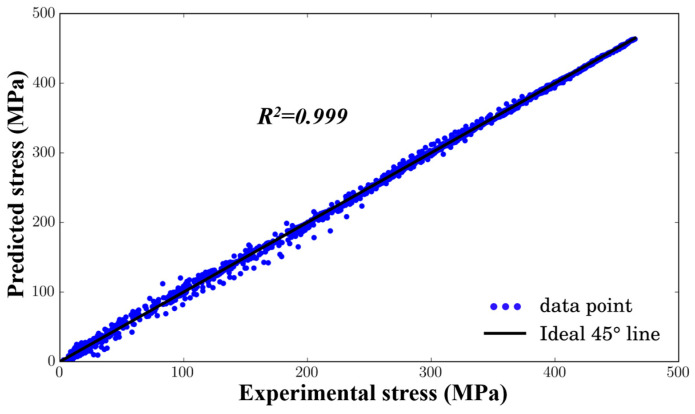
Evaluation of the performance of the ANN model.

Figure 4 shows the comparison between the predicted flow stress from the ANN model and the measured values. It can be seen that the predicted flow stress curve closely matches the experimentally obtained flow stress curve. The *R*^2^, *MRE*, and *RMSE* values are 0.999 (Figure 5), 2.67%, and 3.33, respectively, indicating that the artificial neural network training can effectively predict the deformation behavior of SiC_p_/Al-7.75Fe-1.04V-1.95Si.

(1) $$\begin{array}{c} sig\left(x\right)=\frac{1}{1+{\mathrm{e}}^{-x}} \end{array}$$

(2) $$\begin{array}{c} MRE=\frac{1}{n}\sum_{i=1}^{n}\left|\frac{y_{i}-\hat{y}}{y_{i}}\right|\times 100\% \end{array}$$

(3) $$\begin{array}{c} RMSE=\sqrt{\frac{1}{n}\sum_{i=1}^{n}{\left(y_{i}-\hat{y}\right)}^{2}}\; \end{array}$$

(4) $$\begin{array}{c} R^{2}=1-\frac{\sum_{i}^{n}{\left(y_{i}-\hat{y}\right)}^{2}}{\sum_{i}^{n}{\left(y_{i}-\bar{y}\right)}^{2}}\; \end{array}$$

where *n* is the number of data points used, $\hat{y}$ represents the predicted stress values, $y_{i}$ denotes the experimentally obtained stress values, and $\bar{y}\;$ is the average of the experimentally obtained stress values.

### 3.4. Finite Element Analysis

**Figure 6:**
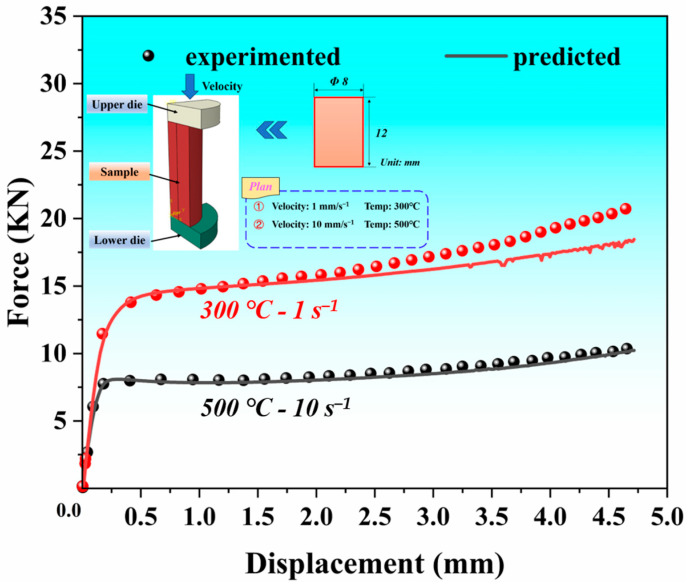
Simulated and experimental force–displacement curves.

From the above analysis, it can be seen that the ANN model can accurately predict the deformation behavior of the SiC_p_/Al-7.75Fe-1.04V-1.95Si composites under different deformation conditions, overcoming the limitations of traditional phenomenological constitutive models. To further apply the ANN constitutive model in finite element calculations, this study integrated the ANN constitutive model into ABAQUS (2022) software, established a thermal compression finite element model, and compared it with experimental results to ensure that the ANN constitutive model is correctly incorporated into the finite element simulations. In this process, the specific implementation code references the work of Olivier Pantale [26]. By analyzing the load–displacement curves from the hot compression process (Figure 6), it can be seen that the load–displacement curve obtained from the numerical simulation shows good agreement with the load–displacement curve observed in the actual hot compression process. This indicates that the finite element model has high computational accuracy and that the ANN constitutive model can be successfully applied to finite element calculations.

**Figure 7:**
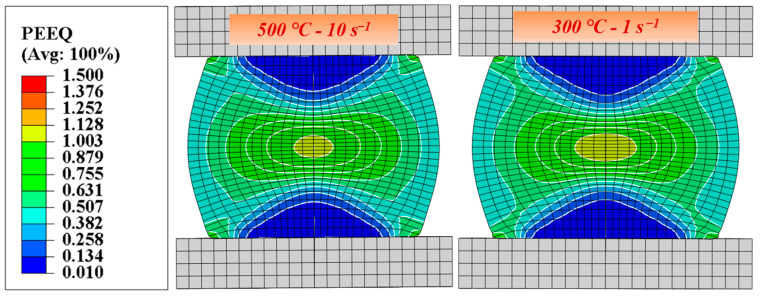
Simulated equivalent plastic strain under different deformation conditions.

Figure 7 shows the distribution of equivalent strain for the SiC_p_/Al-7.75Fe-1.04V-1.95Si composites under different deformation conditions. It can be observed that they all exhibit the same pattern, with uneven strain distribution across different regions, where the central area of the specimen experiences the highest deformation while the regions closer to the die show the least. The main reason for this uneven deformation is the frictional force between the compression die and the specimen. This non-uniform deformation will affect the distribution of SiC particles in the SiC_p_/Al-7.75Fe-1.04V-1.95Si composites to varying degrees. The simulated results agreed well with the microstructural observation in Figure 3.

### 3.5. Thermomechanical Processing Diagram

**Figure 8:**
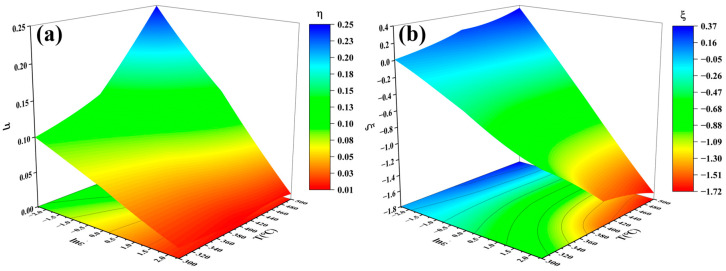
Power dissipation and instability diagrams of the SiC_p_/Al-7.75Fe-1.04V-1.95Si composites: (**a**) power dissipation diagram and (**b**) instability diagram.

**Figure 9:**
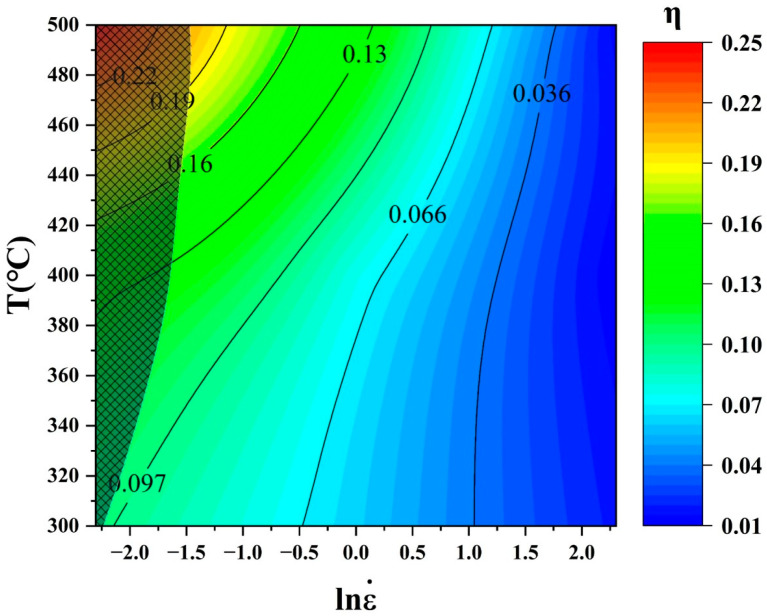
Thermomechanical processing diagram of the SiC_p_/Al-7.75Fe-1.04V-1.95Si composites.

The thermomechanical processing diagram based on the Dynamic Material Model (DMM) [27] is an important research tool for studying material plastic deformation behavior. It helps researchers determine the optimal processing parameter window for the thermal processing of materials. In this paper, a thermomechanical processing diagram for the SiC_p_/Al-7.75Fe-1.04V-1.95Si composites was established based on hot compression data. Figure 8a shows the power dissipation diagram of the SiC_p_/Al-7.75Fe-1.04V-1.95Si composites. When the temperature is between 450 and 500 °C and the strain rate is between 0.1 and 0.3 s^−^^1^, the power dissipation coefficient of the composite material is relatively high. Generally, a higher η value indicates better thermal processing performance of a material. In addition, the instability criterion diagram reveals (Figure 8b) that when the deformation temperature is between 300 and 500 °C and the deformation rate is between 0.1 and 0.2 s^−^^1^, the instability criterion ξ value is positive. This indicates that within this range of deformation parameters, the probability of internal voids, cracks, and fragmentation of reinforcement particles in the material is relatively low. By overlaying the power dissipation diagram with the instability criterion diagram, the thermomechanical processing diagram for the SiC_p_/Al-7.75Fe-1.04V-1.95Si composites can be obtained (Figure 9), with the non-shaded areas representing the instability regions. The thermomechanical processing diagram clearly shows that the optimized processing parameters of the SiC_p_/Al-7.75Fe-1.04V-1.95Si composites are within a deformation temperature of 450–500 °C and a deformation rate of 0.1–0.2 s^−^^1^. This indicates that the material exhibits better thermal processing performance under high temperatures and low strain rates.

## 4. Conclusions

(1) Rheological stress increases rapidly with strain until reaching peak stress, after which it stabilizes or slightly decreases until deformation ceases. Compared with most aluminum matrix composites, the SiC_p_/Al-7.75Fe-1.04V-1.95Si composites exhibited excellent high-temperature resistance. Additionally, SiC reinforcement particles redistribute during the compression process under different deformation parameters.

(2) An artificial neural network model was established to accurately predict the rheological stress behavior of SiC_p_/Al-7.75Fe-1.04V-1.95Si composites. The results showed a high degree of fit between actual and predicted stresses, with an *R*^2^ value reaching 0.999. By incorporating the ANN constitutive model into ABAQUS finite element software (2022), the compression deformation was simulated well, which corresponded to experimental conditions. The simulated load–displacement curves also aligned well with experimental results.

(3) The thermomechanical processing diagram was determined, and the optimal processing region of the SiC_p_/Al-7.75Fe-1.04V-1.95Si composites was found to be a deformation temperature of 450–500 °C and a deformation rate of 0.1–0.2 s^−^^1^.

## Figures and Tables

**Table 1:** Composition of Al-7.75Fe-1.04V-1.95Si alloy (wt.%).

Element	Si	Fe	V	Mg	Mn	Cu	Ti	Zn	Al
	1.95	7.75	1.04	0.013	0.031	0.0041	0.074	0.18	Bal.

## Data Availability

Data is contained within the article.

## References

[1] Idusuyi N., Olayinka J.I. (2019). Dry sliding wear characteristics of aluminium metal matrix composites: A brief overview. J. Mater. Res. Technol..

[2] Chak V., Chattopadhyay H., Dora T.L. (2020). A review on fabrication methods, reinforcements and mechanical properties of aluminum matrix composites. J. Manuf. Process..

[3] Xu S., Yuan L., Wang L., Li J., Xu F., Zheng Z., Shan D., Guo B. (2021). Research on the Secondary Forgeability of High Volume Fraction Whisker Reinforced Aluminum Matrix Composites of Original Squeeze Casting. Materials.

[4] Zou X., Yan H., Chen X. (2016). Compression deformation behavior of semisolid Al_2_O_3np_ reinforced 7075 aluminum matrix composites with high solid fraction. J. Mater. Res..

[5] Haghdadi N., Zarei-Hanzaki A., Abedi H.R. (2012). The flow behavior modeling of cast A356 aluminum alloy at elevated temperatures considering the effect of strain. Mater. Sci. Eng. A.

[6] Seif C.Y., Hage I.S., Hamade R.F. (2020). Incorporating dual BCC/FCC Zerilli-Armstrong and blue brittleness constitutive material models into Oxley’s machining shear zone theory. J. Manuf. Process..

[7] Li X.-Y., Zhang Z.-H., Cheng X.-W., Liu X.-P., Zhang S.-Z., He J.-Y., Wang Q., Liu L.-J. (2022). The investigation on Johnson-Cook model and dynamic mechanical behaviors of ultra-high strength steel M54. Mater. Sci. Eng. A.

[8] Wu C., Chen S., Tang J., Fu D., Teng J., Jiang F. (2023). Hot Workability of the Multi-Size SiC Particle-Reinforced 6013 Aluminum Matrix Composites. Materials.

[9] Pan G., Wang F., Shang C., Wu H., Wu G., Gao J., Wang S., Gao Z., Zhou X., Mao X. (2023). Advances in machine learning- and artificial intelligence-assisted material design of steels. Int. J. Miner. Metall. Mater..

[10] Haghdadi N., Zarei-Hanzaki A., Khalesian A.R., Abedi H.R. (2013). Artificial neural network modeling to predict the hot deformation behavior of an A356 aluminum alloy. Mater. Des..

[11] Huang C., Jia X., Zhang Z. (2018). A Modified Back Propagation Artificial Neural Network Model Based on Genetic Algorithm to Predict the Flow Behavior of 5754 Aluminum Alloy. Materials.

[12] Skinner D.J., Bye R.L., Raybould D., Brown A.M. (1986). Dispersion strengthened Al-Fe-V-Si alloys. Scr. Metall..

[13] Sakata I., Langenbeck S.L. (1983). Elevated Temperature Aluminum Alloys for Aerospace Applications.

[14] Diao E., Fan J., Yang Z., Lv Z., Gao H., Nie J. (2023). Hot Deformation Behavior and Mechanisms of SiC Particle Reinforced Al-Zn-Mg-Cu Alloy Matrix Composites. Materials.

[15] Chen G., Geng H., Ji X., Xu P., Li X., Zhang H. (2023). Investigation of the hot deformation behavior and microstructure evolution of TiB2 +TiAl3/2024Al composite. J. Alloys Compd..

[16] Chen S., Teng J., Luo H., Wang Y., Zhang H. (2017). Hot deformation characteristics and mechanism of PM 8009Al/SiC particle reinforced composites. Mater. Sci. Eng. A.

[17] Zhao T., Zhang B., Zhao F., Zhang Z., Dang X., Ma Y., Cai J., Wang K. (2022). Hot deformation behavior of multilayered Ti/Ni composites during isothermal compression. J. Mater. Res. Technol..

[18] Johnson G.R., Cook W.H.J.E.F.M. A constitutive model and data for metals subjected to large strains, high strain rates and high temperatures. Proceedings of the 7th International Symposium on Ballistics.

[19] Xi N., Fang X., Duan Y., Zhang Q., Huang K. (2022). Wire arc additive manufacturing of Inconel 718: Constitutive modelling and its microstructure basis. J. Manuf. Process..

[20] Fu Z., Gao G., Wang Y., Qiao H., Xiang D., Zhao B. (2022). Research on dynamic mechanical properties and plastic constitutive relation of Ti3Al intermetallic compounds under mechanical-thermal coupling. J. Mater. Res. Technol..

[21] Zhang F., Liu Z., Wang Y., Mao P., Kuang X., Zhang Z., Ju Y., Xu X. (2020). The modified temperature term on Johnson-Cook constitutive model of AZ31 magnesium alloy with {0002} texture. J. Magnes. Alloys.

[22] Yang H., Li M., Bu H., Lu X., Yang H., Qian Z. (2022). Modeling of Flow Stress of As-Rolled 7075 Aluminum Alloy during Hot Deformation by Artificial Neural Network and Application. J. Mater. Eng. Perform..

[23] Wang X., Pan Q., Xiong S., Liu L. (2018). Prediction on hot deformation behavior of spray formed ultra-high strength aluminum alloy—A comparative study using constitutive models. J. Alloys Compd..

[24] Masoudi Nejad R., Sina N., Ma W., Song W., Zhu S.P., Branco R., Macek W., Gholami A. (2024). Artificial neural network based fatigue life assessment of riveted joints in AA2024 aluminum alloy plates and optimization of riveted joints parameters. Int. J. Fatigue.

[25] Fu Y., Shao Z., Liu C., Wang Y., Xu Y., Zhu X. (2022). Modeling the Mechanical Properties of Heat-Treated Mg-Zn-RE-Zr-Ca-Sr Alloys with the Artificial Neural Network and the Regression Model. Crystals.

[26] Pantalé O., Tize Mha P., Tongne A. (2022). Efficient implementation of non-linear flow law using neural network into the Abaqus Explicit FEM code. Finite Elem. Anal. Des..

[27] Prasad Y.V.R.K. (2003). Processing Maps: A Status Report. J. Mater. Eng. Perform..

